# Tattoo pigment in an axillary lymph node simulating metastatic malignant melanoma

**DOI:** 10.1186/1477-7800-2-28

**Published:** 2005-12-01

**Authors:** CM Jack, A Adwani, H Krishnan

**Affiliations:** 1 Breast Unit and Pathology Department Mayday University Hospital, London Road, Croydon, CR7 7YE, Surrey UK

**Keywords:** Tattoo Pigment, Lymphadenopathy, Malignant Melanoma

## Abstract

We report a case of axillary lymphadenopathy thirty years after a decorative tattoo clinically mimicking metastatic melanoma. The importance of relying on histological confirmation of metastatic disease before altering extent of surgery is discussed. The importance of recording presence of decorative tattoos is stressed.

## Background

The presence of lymphadenopathy requires further investigation. Often its presence is explained by a simple viral illness or trauma. Rarer causes are often made apparent by thorough history taking and examination. The need for a biopsy is controversial. We report a case where the answer may have been staring us in the face if we knew where to look. The fact that a tattoo causes lymphadenopathy is well known in the acute phase. This is thought to be due to local inflammation from the initial insult. However, to our knowledge there have been no reports of a palpable node after time delay this long.

## Case report

A 54 year old man presented with a lump in the right axilla of six months duration. The lump was non tender and had not changed in size. He complained of weight loss of 5 kg over the past two months. He denied foreign travel, night sweats, recent injury, cough, or the presence of any other lumps. His past medical history was unremarkable. There was no family history of breast or bowel cancer. The lump was clinically palpable and measured 3 cm. It was firm, non tender, not attached to the skin or deep tissues and was consistent with a clinical diagnosis of axillary lymphadenopathy. The left axilla and supraclavicular fossae were normal. There was no skin lesion in the drainage area of the axilla. Examination of the breasts, chest and abdomen were unremarkable. Haematology, Biochemistry and Chest X-rays were normal. Ultrasound confirmed a benign appearing lymph node with a fatty centre. In view of the size and longstanding nature of the lymph node, an excision biopsy was performed. At surgery the node was firm, suspicious and black in colour.

On histology, the specimen of the lymph node with attached fatty tissue measured 3 × 2 × 0.8 cms. Black discolouration was present on the cut surface.

Microscopic examination of the routine haematoxylin and eosin sections of the lymph node showed preservation of architecture with follicular hyperplasia. Black carbon like pigment was seen lying within the macrophages and dispersed outside them in the sinuses. There was associated fibrosis. Multiple sections did not reveal any evidence of metastatic malignant melanoma.

Immunohistochemical staining for S 100 protein and histochemical stain (Masson's Fontana) was done to further exclude that possibility.

Retrospectively we noted the 30-year old tattoo that the patient had on his right arm.

## Conclusion

Lymphadenopathy refers to nodes that are abnormal in size, consistency or number [1]. There are various classifications of lymphadenopathy, but a simple and clinically useful system is to classify lymphadenopathy as "generalized" if lymph nodes are enlarged in two or more non-contiguous areas or "localized" if only one area is involved. Localised lymphadenopathy of the axilla is suggestive of infections, Cat-scratch disease, Lymphoma, Breast cancer, Silicone implants, Brucellosis and Melanoma. The presence or otherwise of a tattoo may not be noted in history taking for lymphadenopathy [2]. Little information exists to suggest that a specific diagnosis can be based on node size. However, in one series of 213 adults with unexplained lymphadenopathy, no patient with a lymph node smaller than 1 cm^2 ^had cancer, while cancer was present in 8 percent of those with nodes from 1 cm^2 ^to 2.25 cm^2 ^in size, and in 38 percent of those with nodes larger than 2.25 cm^2 ^[3]. In children, lymph nodes larger than 2 cm in diameter (along with an abnormal chest radiograph and the absence of ear, nose and throat symptoms) were predictive of granulomatous diseases (i.e. tuberculosis, cat-scratch disease or sarcoidosis) or cancer (predominantly lymphomas) [4].

The fact that a tattoo causes lymphadenopathy is well known in the acute phase due to local inflammation and probably resolves spontaneously. The natural history of tattoo is well documented. The tattoo ink particles may range from 2–400 nm and are most commonly 40 nm. They are initially found within large phagosomes in the cytoplasm of keratinocytes, phagocytic cells including fibroblasts, macrophages and mast cells. The skin layers initially appear homogenised but at one month, the basement membrane is reforming and aggregates are present within basal cells. At 2–3 months and at 40 years, ink particles are only found in dermal fibroblasts surrounded by a network of connective tissue that entraps and immobilises the cell. The tattoo may appear blurred with time due to ink movement into the deep dermis. Eventually the tattoo ink appears in the regional lymph nodes.

This is thought to be due to local inflammation from the initial insult. However, to our knowledge there have been no reports of a palpable node after time delay this long. The dye used in skin tattooing is carbon based.

The movement of dye through the lymph channels forms the basis of sentinel node biopsy. Complications of lymph node biopsy are reported as scaring, blood loss, infection and more rarely nerve damage and lymphoedema. The question remains whether it was necessary to biopsy this lymph node or was the presence of the tattoo enough to give reason for the enlarged node. In this instance the co factor of the weight loss meant that leaving the node would not be reasonable.

Anderson [5] and Moehrle [6] reported that tattoo pigments in Lymph nodes can mimic metastatic malignant melanoma, but do not comment on age of the decorative tattoo. Such pigmentation in patients with malignant melanoma can look metastatic and may prompt the surgeon to proceed to complete nodal dissection. Nodal dissection should be delayed till conclusive histological diagnosis is made [7].

**Figure 1:**
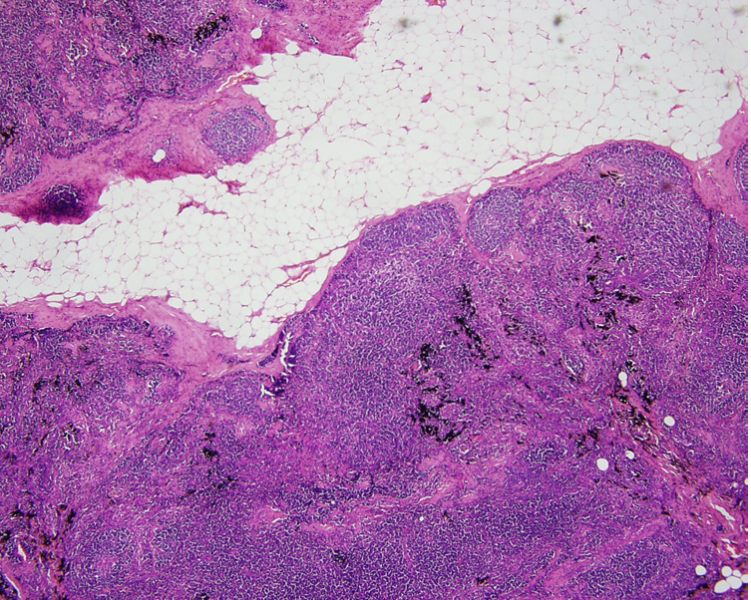
Lymph node with preserved architecture and the pigment. H&E × 100

**Figure 2:**
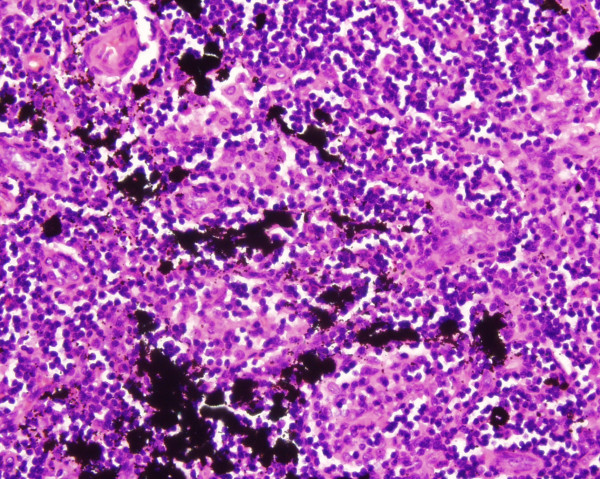
The dark granular carbon pigment located in the sinuses. H&E × 400

Migration of the carbon pigment through the lymphatics is usually seen in the hilar lymph nodes draining the lungs. The main differential diagnosis in our case would be metastatic malignant melanoma. This was excluded by the careful examination of the H&E sections for tumour cells (Figure 1, 2) and employing special stains. Immunohistochemical staining for S 100 protein is a very sensitive marker for melanoma cells and a Masson's Fontana stain helps to differentiate melanin pigment from carbon pigment.

Sentinel lymph node biopsy is becoming more common in Melanoma and Breast cancer. History taking and examination should include presence, site, age and colour of decorative tattoos especially in the drainage areas to the axilla. History of removal of tattoos is also important as nodes may persist for several years. Raising awareness of this problem among surgeons and pathologists treating malignant melanoma is important. Investigation of axillary Lymphadenopathy should include tattoos in the drainage areas as a probable cause.

## References

[B1] Goroll AH, May LA, Mulley AG (1987). Primary care medicine: office evaluation and management of the adult patient.

[B2] Ferrer R Lymphadenopathy: Differential Diagnosis and Evaluation. American Family Physician.

[B3] Pangalis GA, Vassilakopoulos TP, Boussiotis VA, Fessas P (1993). Clinical approach to Lymphadenopathy. Semin Oncol.

[B4] Slap GB, Brooks JS, Schwartz JS (1984). When to perform biopsies of enlarged peripheral lymph nodes in young patients. JAMA.

[B5] Anderson LL, Cardone JS, McCollough ML, Grabski WJ (1996). Tattoo pigment mimicking malignant melanoma. Dermatological Surgery.

[B6] Moehrle M, Blaheta HJ, Ruck P (2001). Tattoo pigment mimics positive sentinel lymph node in melanoma. Dermatology.

[B7] Friedman T, Westreich M, Mozes SN (2003). Tattoo Pigment in Lymph Nodes Mimicking Metastatic Malignant Melanoma. Plast Reconstructive Surg.

